# Experimental Validation of Slip-Forming Using Ultrasonic Sensors

**DOI:** 10.3390/s19225053

**Published:** 2019-11-19

**Authors:** Hyejin Yoon, Young Jin Kim, Won Jong Chin, Jun Won Kang, Hyun-Moo Koh

**Affiliations:** 1 Seismic Safety Research Center, Korea Institute of Civil Engineering and Building Technology, 283 Goyangdae-ro, Ilsanseo-gu, Goyang-si, Gyeonggi-do 10223, Korea; hiyoon@kict.re.kr (H.Y.); yjkim@kict.re.kr (Y.J.K.); wjchin@kict.re.kr (W.J.C.); 2 Department of Civil Engineering, Hongik University, 94 Wausan-ro, Mapo-gu, Seoul 04066, Korea; 3 Department of Civil and Environmental Engineering, Seoul National University, 1 Gwanak-ro, Gwanak-gu, Seoul 08826, Korea; hmkoh@snu.ac.kr

**Keywords:** slip-form, ultrasonic sensors, surface wave velocity, compressive strength

## Abstract

Slip-forming in concrete construction enables the continuous placement of concrete using a climbing form, the efficiency of which depends on appropriate slip-up timing. This implies the importance of knowing accurately the development of concrete strength over time, which has been assessed manually to date in construction fields. This paper presents a method for automating the slip-forming process by determining the optimal slip-up time using the in-situ strength of concrete. The strength of concrete is evaluated by a formula relating the strength to the surface wave velocity measured with ultrasonic sensors. Specifically, this study validates the applicability of the slip-form system with ultrasonic sensors for continuously monitoring the hardening of concrete through its application in several construction sites. To this end, a slip-form system with a pair of ultrasonic modules at the bottom of the panel was tested and the time variation of surface wave velocity in the concrete material was monitored during the slip-forming process. The results show that the proposed method can provide the optimal slip-up time of the form to automate the slip-forming process. This approach is expected to apply to other construction technologies that required the continuous monitoring of concrete strength for construction efficiency as well as quality maintenance.

## 1. Introduction

Slip-forming in concrete construction shortens the construction period significantly by enabling an automated form, moving at a rate of 2 to 4 m per day with the 24-h continuous placing of concrete [1]. The form can move to the next position once the concrete inside the form develops sufficient early strength [2]. Although slip-forming has become the main construction method for tall concrete structures such as bridges, towers, and buildings, there is as yet no standard for specifying, quantitatively, the proper time to lift the slip-form. ACI 347-14 [3] suggests that the compressive strength of the concrete in the form be checked frequently by a skilled technician. However, there is no consensus among engineers about the desirable strength level of concrete for slip-form stripping [4]. Based on field experience, previous studies proposed 0.2 MPa as a sufficient level of compressive strength for form lifting and even reduced this value to 0.1 MPa for more rapid lifting [4]. Taking into account the fact that the pressure by the overlying concrete layer inside the form is critical in slip-forming, Reichverger and Jaegermann said that the pressure exerted on the exposed casting by the overlying concrete layer inside the form should be limited to a maximum of 0.025 MPa [4]. Reichverger also reported that the optimal condition for moving the slip-form is achieved when the compressive strength of the concrete in the remolding zone inside the form lies between 0.2 and 0.3 MPa [2]. However, since it is difficult to monitor the in-situ strength of fresh concrete continuously, the compressive strength of concrete on-site is often assessed manually by introducing, for example, a cone penetration method to the concrete that has been poured in the slip-form.

**Figure 1:**
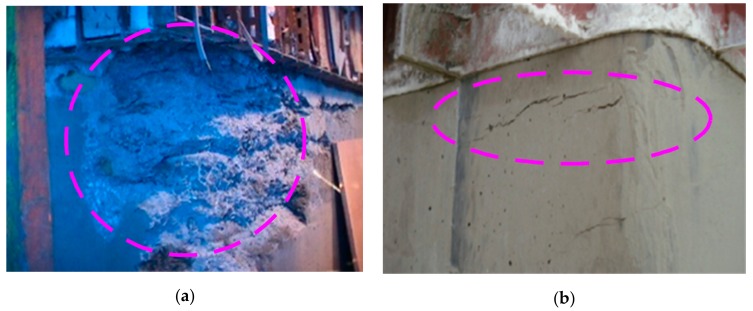
Results of improper form lifting: (**a**) premature form removal and (**b**) delayed form removal [5].

Figure 1 shows the surface of concrete separated from the formwork after improper form lifting. If the concrete is exposed to the weather too early by the fast-climbing of the slip-form, the concrete may deform excessively as shown in Figure 1a or may fail to develop sufficient strength. On the other hand, if the climbing is too slow, the slip-form may experience deformation, as shown in Figure 1b, due to the excessive lifting force required to subdue the adhesion between the form and the concrete, or the stability of the form may be degraded. These examples stress the importance of monitoring the compressive strength of concrete and determining the proper moving time of the slip-form.

In recognition of this need, various nondestructive evaluation methods, such as embedded piezoelectric transducers [6,7,8] and electro-mechanical impedance methods [9,10,11], have been developed to monitor the early-age strength of concrete during construction. In particular, the ultrasonic wave velocity method, which uses surface waves among various wave components, has been demonstrated to be effective for evaluating the hardening of concrete with various levels of embedment [12,13,14,15].

Previous studies by the authors [16,17] suggested a surface wave velocity method to evaluate the early-age compressive strength of concrete inside the form. As a main result of the studies, an empirical formula for predicting the early-age compressive strength of concrete was developed based on the measured data, including a temperature coefficient to account for the seasonal effect on construction. The present study stands on the ground of the previous study to propose a slip-forming system with ultrasonic sensors for measuring surface waves. This paper presents the application results of the ultrasonic method for construction with different curing temperatures and discusses practical considerations for obtaining reliable estimates of the compressive strength of concrete.

## 2. Evaluation of the Compressive Strength of Concrete in Early Ages

This chapter discusses the relationship between the compressive strength of concrete and Rayleigh wave velocity, especially in the short time after the casting of the concrete. Rayleigh waves, a type of elastic surface waves, travel along the shallow part of a solid medium. In a homogeneous and isotropic medium, the velocity of Rayleigh waves is known to be related to the dynamic modulus of elasticity $E_{d}$, the mass density ρ, and Poisson’s ratio ν of the medium as follows [18].

(1) $$V_{R}=\frac{0.87+1.12\nu }{1+\nu }\sqrt{\frac{E_{d}}{2(1+\nu )\rho }}$$

From Equation (1), the dynamic modulus of elasticity can be formally expressed by the Rayleigh wave velocity as:

(2) $$E_{d}=f(\rho ,\;\nu )V_{R}^{2}$$

where f(ρ, ν) denotes a function of ρ and ν. With the consideration of the strain-rate-dependent behavior of concrete, former studies [19,20] have shown from laboratory tests that the static modulus of elasticity of concrete can be expressed by its dynamic modulus as:

(3) $$E_{c}=\alpha E_{d}$$

where α is a proportional constant that can be determined from experiments. Several design codes for concrete structures relate the static modulus of elasticity of concrete to its compressive strength. For example, ACI318-14 [21] prescribes the relationship as in Equation (4), and Korean Highway Bridge Design Code (Limit State Design) [22] presents the relationship as in Equation (5):

(4) $$E_{c}=\rho^{1.5}0.043f_{c}^{\frac{1}{2}}$$

(5) $$E_{c}=\rho^{1.5}0.077f_{c}^{\frac{1}{3}}$$

**Figure 2:**
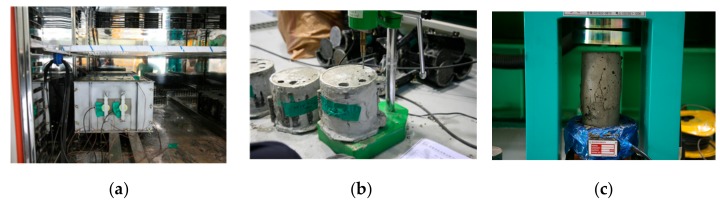
Experiments for in-situ concrete at an early age: (**a**) specimen for the surface wave velocity measurement, (**b**) specimen for the penetration resistance test, and (**c**) specimen for the cylindrical compressive strength test.

**Figure 3:**
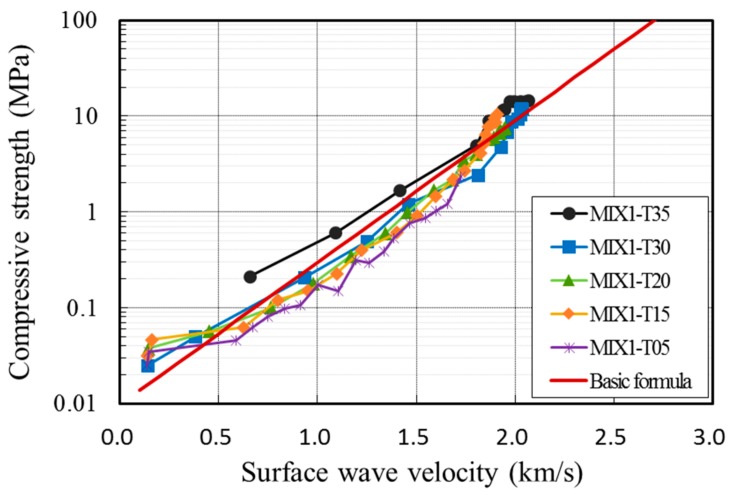
Correlation between compressive strength and surface wave velocity for early-aged concrete [17]; the graph of Equation (8) is compared with test data for concrete cylinders in various temperatures.

Equations (4) and (5) imply that the compressive strength of concrete can be formally written as a function of $E_{c}$ as:

(6) $$f_{c}=g(\rho )E_{c}^{2\sim 3}$$

in which g(ρ) denotes a function of density. By combining Equations (2), (3) and (6), one can represent the compressive strength of concrete in a power function form as:

(7) $$f_{c}=h(\rho ,\;\nu )V_{R}^{\alpha }$$

where α is the power, and h(ρ, ν) denotes a function of ρ and ν. There have been several works of literature that discuss the power function form to evaluate the compressive strength of concrete using the velocity of P or Rayleigh waves [23,24,25]. Along with such developments, it has also been shown in recent studies that an exponential form of the function can represent the relationship better, especially for early-aged concrete [12,26,27]. In particular, Ref. [17] proposed an empirical formula to predict the compressive strength of concrete in early ages using the surface wave velocities measured in a set of concrete specimens. The strength formula reads:

(8) $$f_{c}=0.0098ke^{3.412V_{R}}$$

where $f_{c}$ is the compressive strength of concrete in MPa, $V_{R}$ is the surface wave velocity in km/s, and k is the dimensionless thermal coefficient that accounts for the effect of temperature during construction. Table 1 presents the value of k for three groups of temperatures between 5 °C and 35 °C and surface wave velocities for the compressive strengths of 0.2 Mpa and 0.3 Mpa. Figure 2 shows laboratory tests for the compressive strength and the penetration resistance of concrete during the first 24 h after casting. The surface wave velocity was measured for each of the specimens and was correlated with the compressive strength of concrete to yield Equation (8). Figure 3 shows the feasibility of Equation (8) in representing the correlation between them.

## 3. Slip-Forming with Ultrasonic Sensors

**Figure 4:**
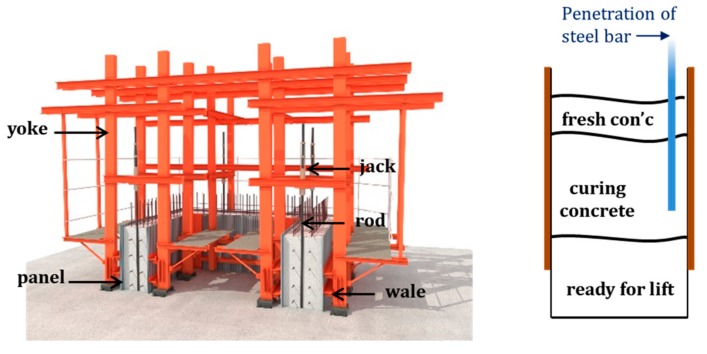
Schematic diagram of a slip-form system.

A slip-form system for concrete construction consists of various components such as a hydraulic jack, steel rods, platforms, etc. Figure 4 shows the schematic diagram of a slip-form system. In the slip-form system, the formwork is supported by vertical rods embedded in the concrete core and lifted by hydraulic jacks connected to yokes. In the field operation, the lifting time of the slip-form has typically been determined by technicians based on professional experience or qualitative evaluation using the probing apparatus. Such slip-forming operations make it difficult to ensure the consistency of concrete quality during construction. In this work, the feasibility of an ultrasonic sensor module for measuring surface wave velocity was investigated for the slip-forming operation.

**Figure 5:**
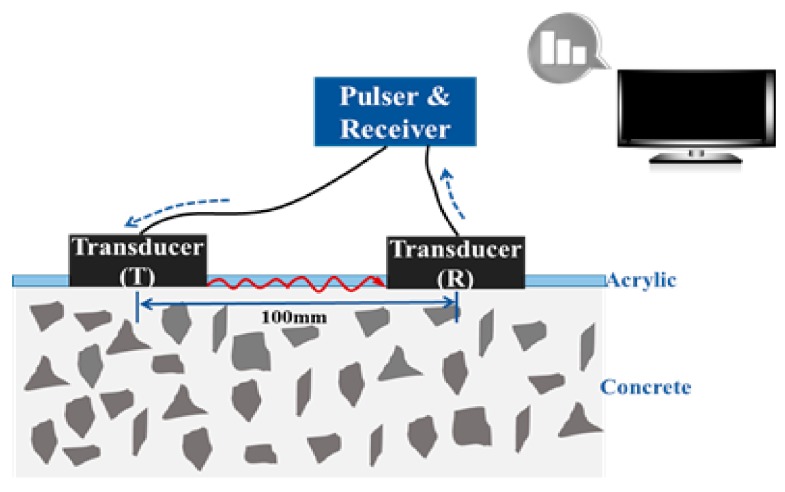
Schematic of an ultrasonic module for measuring surface wave velocity [17].

Figure 5 shows the schematic set-up of the ultrasonic module attached to a concrete surface for measuring the surface wave velocity of early-aged concrete inside the form. The module was composed of two ultrasonic transducers, a pulser-receiver device, a data acquisition system, and a display device. The two ultrasonic transducers were placed 100 mm apart from each other on the bottom of the form. The pulser sent a short-duration voltage signal to one transducer, causing it to vibrate at its resonant frequency. The surface waves generated from the transducer travelled to the other transducer through the surface of concrete inside the form. The surface wave velocity was calculated using the arrival time of the waves, which could be determined by a continuous wavelet transform (CWT) [12,17]. The CWT showed the spectrum of waves in both the frequency and time domains, and therefore, could estimate the travel time of propagating waves in a frequency range of interest effectively. The method of using CWT was more efficient and systematic than just using the arrival time of the first peak, which was difficult to distinguish from complex waveform data [28,29,30].

**Figure 6:**
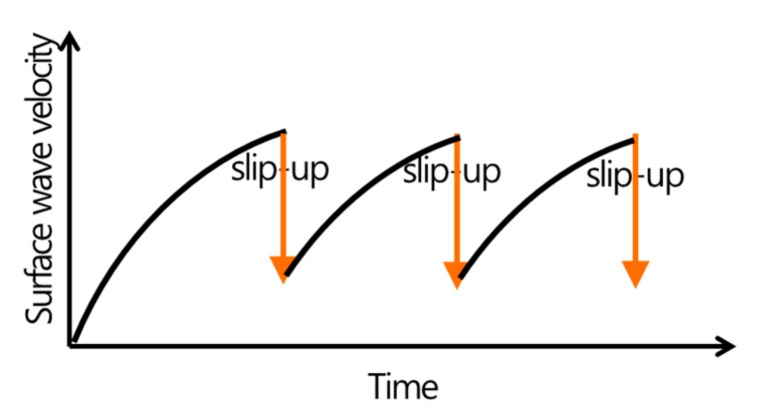
Slip-forming sequence using the surface wave velocity in early-aged concrete.

Figure 6 shows a slip-forming sequence using the surface wave velocity data collected from the ultrasonic module. The surface wave velocity was continuously measured from the first casting of concrete. The velocity increased as the concrete hardened and the slip-form could be moved up when the strength of concrete reached 0.2~0.3 MPa, a practical range for slip-forming in concrete construction. The velocities for the strength range can be calculated using Equation (8) and are tabulated in Table 1. After the slip-form was lifted and the new concrete was cast, the surface wave velocity measured for the new layer dropped down sharply, as shown in Figure 6. The slip-forming process was repeated until the concrete casting was completed. In this work, the surface wave velocity in early-aged concrete at which the form-lifting was allowed was proposed to be in the range of 1.0 km/s to 1.4 km/s.

**Figure 7:**
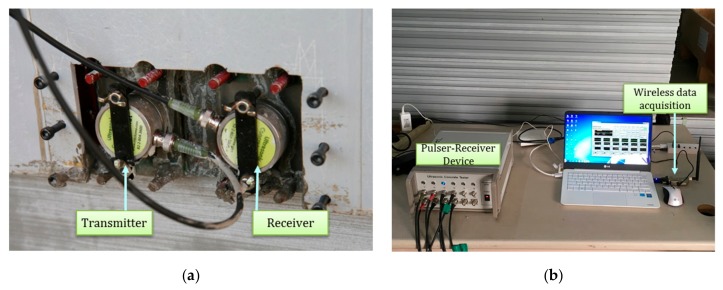
Measurement setup for slip-forming: (**a**) ultrasonic transducers, (**b**) data acquisition system [5]; the models of the ultrasonic transducer, the pulser-receiver, and the wireless data acquisition unit are CT-1010, Ultracon-3030, and MKDQ-710 of MKC Korea, respectively.

**Figure 8:**
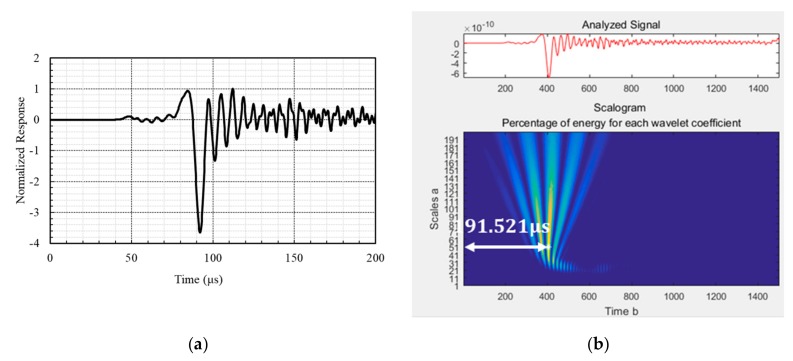
Measured time history of surface waves and the contour plot of the Morlet wavelet for the propagating waves [17]: (**a**) time history and (**b**) travel time calculating with the Morlet wavelet.

Figure 7 shows the ultrasonic module developed in this study. The module had a piezoelectric ultrasonic transducer pair mounted on the form panel, as shown in Figure 7a. The nominal frequency of the transducer was 50 kHz. The ultrasonic sensors were installed 100 mm apart on the panel on the same side of the concrete wall. A pulser-receiver device was used to generate an electric signal with 600 V or 1200 V, which was transformed into an ultrasonic signal by the transducer. The pulser-receiver also collected the incoming signal with a sampling rate of 1 to 10 MHz. The sampling rate in this study was 2.5 MHz. The wireless data acquisition unit shown in Figure 7b transferred the measured signal to a computer. Figure 8 shows a sample of the measured waveform and its spectrum obtained from CWT. The travel time of the surface waves was identified as the time of the largest peak in the time-frequency spectrum of the measured waveform.

## 4. Validation of Slip-Forming Using Ultrasonic Sensors 

For validating the applicability of the ultrasonic sensor system to the slip-forming of concrete, the system was applied to the construction of full-scale concrete towers using a slip-forming method. The test considered different temperature conditions to verify the system’s applicability to various seasons.

### 4.1. Application of the Slip-Forming System at Moderate Temperature

**Figure 9:**
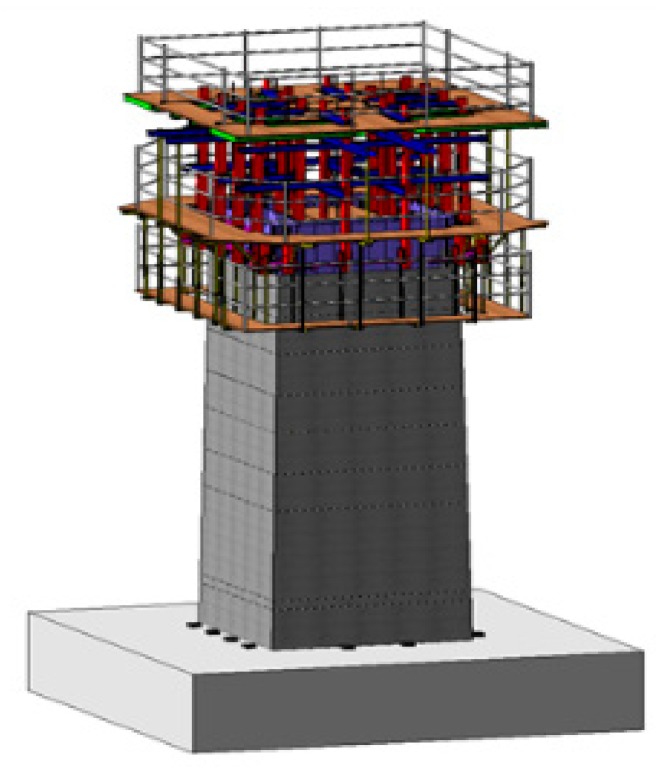
Configuration of the slip-form on a single concrete tower.

**Figure 10:**
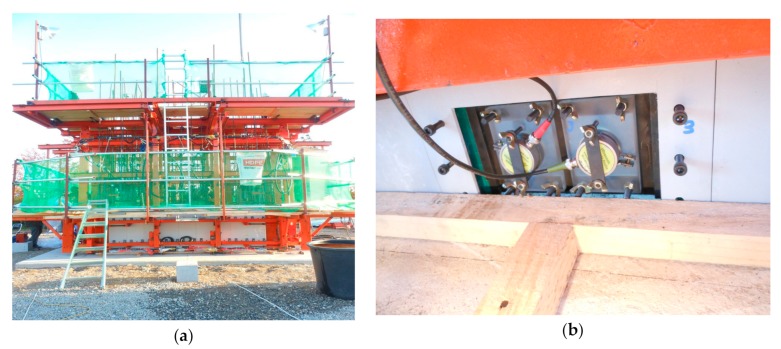
Slip-form system for the single concrete tower: (**a**) the assembled slip-form system and (**b**) the ultrasonic module installed at the bottom of a Glass Fiber Reinforced Polymer (GFRP) panel.

The first application was on a 10 m high single concrete tower with a rectangular hollow section. The mix proportion and design strength of concrete are listed in Table 2. Figure 9 shows the configuration of the slip-form on the concrete tower. The cross-section of the tower was 4.0 m × 4.0 m with a thickness of 0.6 m at the foot, and the cross-section was 3.77 m × 3.6 m at the top, where the thickness of three sides was 0.6 m and the remaining one was 0.5 m. In general, steel panels are preferred for the slip-form. However, several studies reported that the form panel made of Glass Fiber Reinforced Polymer (GFRP) developed better performance than its steel counterpart [31,32]. Therefore, the slip-form developed in this study combined both steel and GFRP panels. Steel panels were used for inner forms, whereas the GFRP panels were used for outer forms except for the one on the side with sectional change. Figure 10 shows the slip-form system assembled on-site and the ultrasonic module installed at the bottom of the GFRP panel.

**Figure 11:**
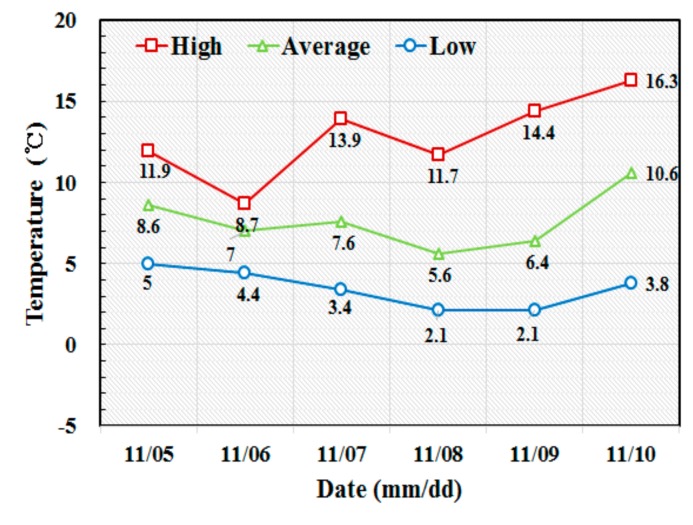
Atmospheric temperature measured during the construction of the single concrete tower.

**Figure 12:**
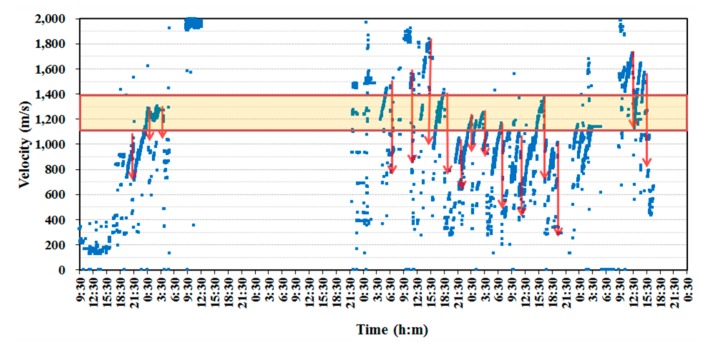
Surface wave velocity monitored by the ultrasonic slip-forming system during the construction of the single concrete tower.

In this study, it was determined that the slip-form would be lifted when the strength of the cast concrete reached the required range of 0.2 to 0.3 Mpa. The concrete casting for the building of the tower was done in early November when the temperature ranged between a minimum of 2.1 °C and a maximum of 16.3 °C. The average daily temperature was between 5.6 °C and 10.6 °C, as shown in Figure 11. Therefore, the temperature groups B and C in Table 1 were considered for the application of Equation (8) for the slip-forming. With the temperature coefficients reflected in the calculation of the surface wave velocity, the lift-up of the slip-form system was done when the wave velocity reached the range of 1.113 to 1.399 km/s. The surface wave velocity was measured continuously by the ultrasonic module at the bottom of the GFRP panel from the start of concrete casting to the end of the tower erection except for a specific period. The slip-form system was lifted to the next position for the first time 11 h after the initial casting based on the measured surface wave velocity. As shown in Figure 12, the time history of the monitored surface wave velocity exhibited a succession of steady increases and sudden drops. The wave velocity started to increase after a new concrete layer was placed. Once the velocity reached a value between 1.113 and 1.399 km/s, indicating the development of the required strength of concrete, the slip-form system was raised to expose the hardened concrete layer to the air and cast a new concrete layer on top. Then, the surface wave velocity sharply dropped, as indicated by downward arrows in Figure 12, since the wave velocity was relatively lower in the upper layer than in the hardened bottom layer. The velocity data could not be measured on the second and third days after the first casting, because of a temporary operation outage due to an unexpected problem with the slip-form system.

**Figure 13:**
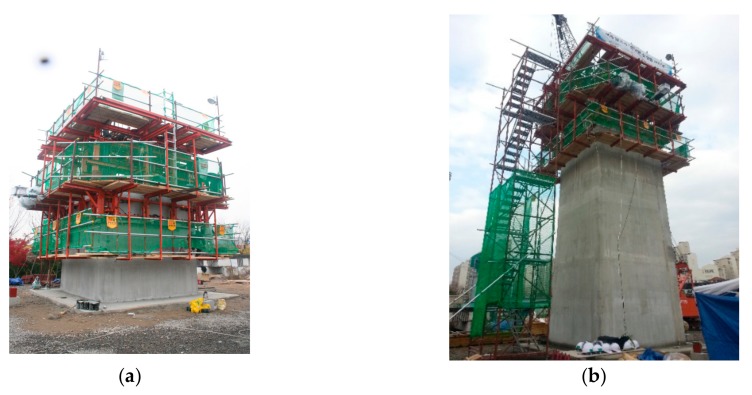
Construction of the single concrete tower: (**a**) during construction and (**b**) at completion.

Figure 13 shows the developed slip-forming system and the concrete tower during the construction. The adoption of the ultrasonic module allowed for the automated slip-forming and fast construction of the 10 m high concrete tower without relying on the assessment of concrete strength by a cone penetration method. The installation of the ultrasonic module at the bottom of the GFRP panel did not provoke any leakage of concrete. As a result, the condition of the concrete surface was good in all the sections of the tower. The implementation confirms the field applicability of the surface wave velocity method using the developed ultrasonic module for the slip-forming of concrete structures.

### 4.2. Application of the Slip-Forming System at Low Temperature

**Figure 14:**
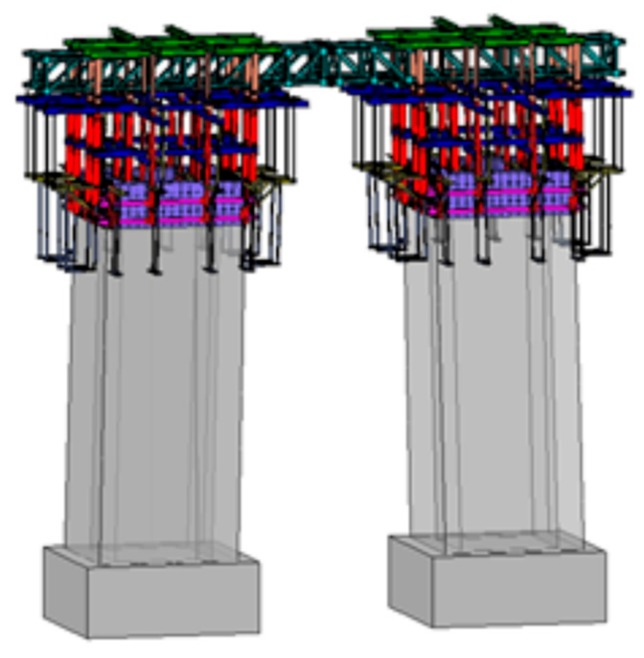
Configuration of slip-forms on a twin concrete tower.

**Figure 15:**
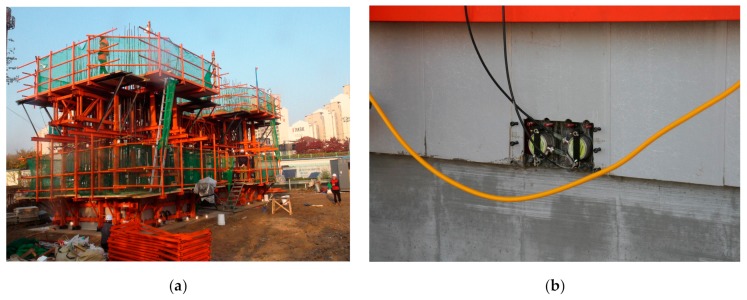
Slip-form system for twin-shaft concrete tower: (**a**) assembled slip-form system and (**b**) ultrasonic module installed at the bottom of a GFRP panel.

The second application was on a 10 m high twin-shaft concrete tower with a rectangular hollow section, commonly adopted in cable-supporting bridges. Figure 14 shows the configuration of the slip-forms on the concrete tower. The cross-section size of the shafts and the distance between the two shafts decreased toward the top. The cross-section of a shaft was 4.0 m × 4.0 m with a thickness of 0.6 m at the foot, and the cross-section was 4.0 m × 3.67 m at the top, where the thickness of three sides was 0.6 m and the remaining one was 0.55 m. The distance between the shafts was 6 m on the ground and 5.5 m at the top. For adjusting the distance between the shafts, a horizontal lattice beam connecting the slip-form system to each shaft was installed. The slip-form used steel and GFRP panels in this case as well. Steel panels were used for the inner forms, whereas GFRP panels were used for the outer forms except for the one on the side with sectional change. Figure 15 shows the slip-form system assembled on-site for the twin-shaft concrete tower and the ultrasonic module installed at the bottom of the GFRP panel. Table 3 presents the mix proportion of concrete used for the tower.

**Figure 16:**
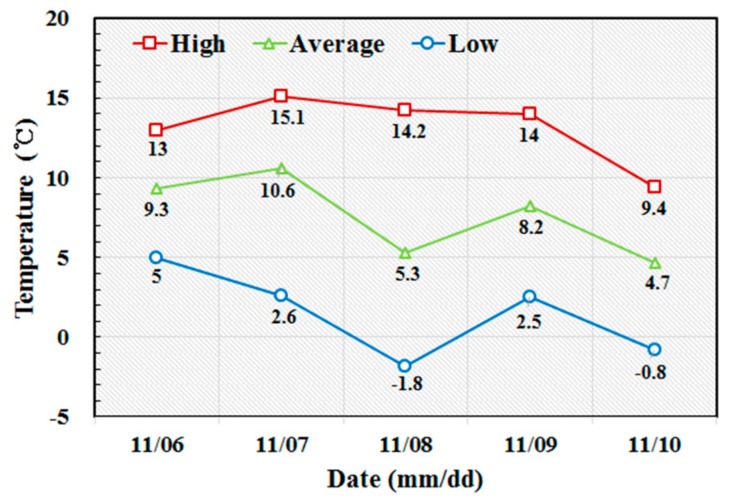
Atmospheric temperature measured during the construction of the twin-shaft concrete tower.

**Figure 17:**
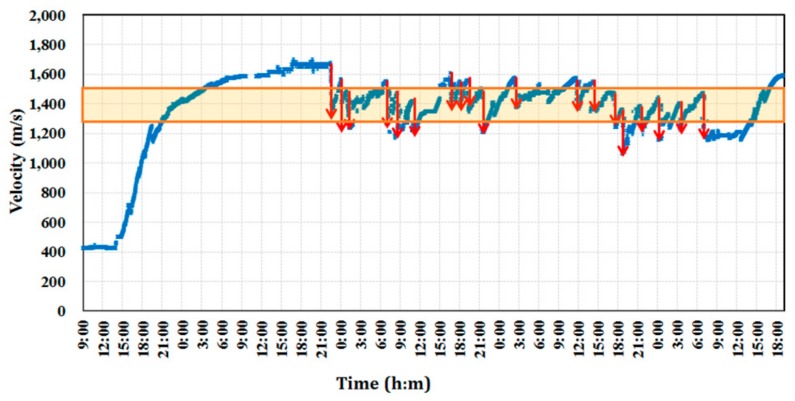
Surface wave velocity monitored by the ultrasonic slip-forming system during the construction of the twin concrete tower.

Concrete casting for the twin tower was done in mid-November when the temperature ranged between a minimum of −1.8 °C and a maximum of 15.1 °C. The average daily temperature was between 4.7 and 10.6 °C as shown in Figure 16. The lower temperature was below the minimum temperature of 5 °C considered in deriving the relationship between the compressive strength of concrete and surface wave velocity. For using Equation (8), the thermal coefficient k was extrapolated and the value of 0.166 was applied for −2 °C. Therefore, surface wave velocity ranging from 1.280 to 1.531 km/s, corresponding to a compressive strength range of 0.2~0.3 MPa was adopted to allow for the slip-up of the forms. Figure 17 shows the values of the surface wave velocity measured continuously by the ultrasonic module in the slip-form from the start of concrete casting to the end of tower erection. The first slip-up was carried out 11 h after the initial casting when the surface wave velocity reached 1280 km/s. The time history of the monitored surface wave velocity shows a succession of steady increases and sudden drops in this case as well. Once the surface wave velocity reached a value between 1.280 and 1.531 km/s for the bottom concrete layer, the slip-form system was raised for the casting of the upper concrete layer. The downward arrows in Figure 17 show the sudden drop of the wave velocity, as it was measured on a new bottom concrete layer after the lifting of the slip-form.

**Figure 18:**
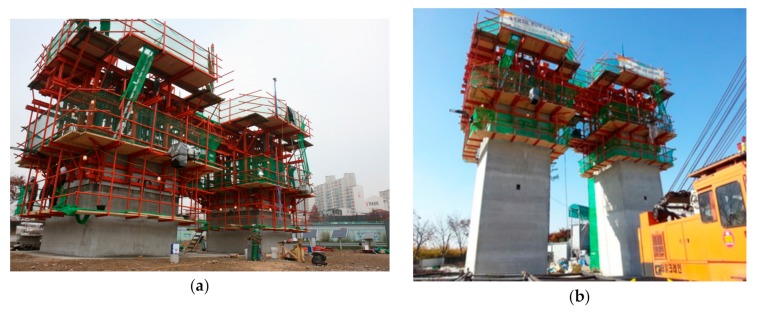
Construction of the twin concrete tower: (**a**) during construction and (**b**) at completion.

Figure 18 shows the erected twin-shaft tower with the developed slip-forming system. Again, the adoption of the ultrasonic module allowed for the automated slip-forming and the fast completion of the 10 m high twin-shaft concrete tower. The satisfactory concrete surface was obtained in all the sections using the slip-forming method. The implementation confirms the field applicability of the surface wave velocity method for the slip-forming of concrete structures even for temperatures falling outside of the originally considered range. The developed ultrasonic sensor module can be further improved by using a piezo-composite transducer, which allows a lower voltage signal to generate surface waves [33,34].

## 5. Conclusions

This study investigated the applicability of a surface wave velocity method to the slip-forming of concrete structures. The wave velocity could be measured by a developed ultrasonic sensor module mounted on the slip-form. The slip-form system was applied to the construction of two full-scale concrete towers under different temperature conditions. The implementation showed that the surface wave velocity could provide a quantitative and objective indicator for the compressive strength of early-aged concrete inside the form. As a result, the surface wave velocity method with the ultrasonic module enabled the automation of slip-forming without relying on the subjective assessment of technicians on proper slip-up time. The ultrasonic module mounted on the slip-form did not provoke any leakage of concrete during casting and can be easily dismantled after the construction. With the timely slip-forming by the developed ultrasonic measurement system, the field implementation could yield an excellent quality to the concrete surface of the towers. Although the relationship between the compressive strength of concrete and the surface wave velocity was established for temperatures from 5 to 35 °C, it is applicable to temperatures outside of this range by an adjustment to the thermal coefficient. Further research is needed to investigate the applicability of the smart slip-forming method for a wider range of temperatures considering extreme weather conditions during construction.

## Figures and Tables

**Table 1:** Thermal coefficient k in Equation (8) for three groups of temperatures.

Temperature (°C)	*k*	Velocity, *V_R_* (km/s)	
		*f_c_* = 0.2 MPa	*f_c_* = 0.3 MPa
30~35 (Group A)	0.579	1.044	1.163
15~20 (Group B)	0.458	1.113	1.232
5 (Group C)	0.259	1.280	1.399

**Table 2:** Mix proportions of the single tower concrete.

W/C * (%)		35.4
S/A ** (%)		46
Unit weight (kg/m^3^)	Fly ash	−
	Super-plasticizer	4.75
	Cement	475
	Sand	760
	Gravel	935
Design strength (MPa)		40

* water to cement ratio, ** sand to aggregate ratio.

**Table 3:** Mix proportions of the twin tower concrete.

W/C * (%)		35.4
S/A ** (%)		46
Unit weight (kg/m^3^)	Fly ash	−
	Super-plasticizer	4.75
	Cement	475
	Sand	760
	Gravel	935
Design strength (MPa)		40

* water to cement ratio, ** sand to aggregate ratio.
